# Introducing *Journal of Medical Case Reports*

**DOI:** 10.1186/1752-1947-1-1

**Published:** 2007-02-02

**Authors:** Michael Kidd, Charlotte Hubbard

**Affiliations:** 1 Discipline of General Practice, The University of Sydney, 37A Booth Street, Balmain, Sydney, NSW 2041, Australia; 2 BioMed Central, Middlesex House, 34-42 Cleveland Street, London W1T 4LB, UK

## 

A case report provides important and detailed information about an individual, which is often lost in larger studies. Moreover, case reports can serve as an early warning signal for the adverse effects of new medications, or the presentations of new and emerging diseases. Although a number of medical journals publish case reports, these articles are underrepresented in the literature, and there has not been a journal specifically devoted to these valuable reports. *Journal of Medical Case Reports *fills this void. It is an online, open access journal dedicated to the publication of high quality case reports, and aims to contribute to the expansion of current medical knowledge.

Since antiquity, clinicians have learnt from their more experienced peers as well as from their own work with their individual patients [1]. Accurate recounting of clinical experience continues to be essential to the progress of medicine. In recent years, however, case reports have become a casualty of the pursuit of the impact factor. Case reports tend to have low citation value and therefore can negatively affect a journal's impact factor, and prevalence of these articles in high impact journals has been reported as being in decline [2]. With *Journal of Medical Case Reports *we intend to create a dedicated home for these articles, which have the potential to contribute to and change clinical practice.

All content in *Journal of Medical Case Reports *is open access, and therefore articles are freely and immediately available online, making this journal an important global resource. In line with our open access policy, authors retain the copyright of their work, allowing them to distribute their article as they please. In addition, all articles are archived in PubMed, PubMed Central, and also in repositories at the University of Potsdam in Germany, at INIST in France and in e-Depot, the National Library of the Netherlands' digital archive of all electronic publications. As the cost of publishing, maintaining and archiving research articles is not recouped through subscription charges, a standard article-processing charge (APC) is levied on articles that are accepted for publication. The APC is a flat charge, and no additional costs are incurred, for example, by the inclusion of colour figures.

All case reports published in *Journal of Medical Case Reports *will be aggregated into a structured case reports database in due course. The case reports database, currently in development, will also draw content from other sources, making it possible to search for patterns of drug reactions, or demographic data and disease information, across multiple case reports. We believe the database will become an important resource for clinicians, as well as academic, commercial and legislative organisations that monitor medication side effects, new and emerging diseases and changes in patterns of known diseases.

*Journal of Medical Case Reports *invites submission of original case reports that expand the field of general medical knowledge. Case reports should be short (no more than 2000 words with a maximum of 10 references and 3 figures). We invite concise articles that fall into one of the following seven categories:

1. Unreported or unusual side effects or adverse interactions involving medications

2. Unexpected or unusual presentations of a disease

3. New associations or variations in disease processes

4. Presentations, diagnoses and/or management of new and emerging diseases

5. An unexpected association between diseases or symptoms

6. An unexpected event in the course of observing or treating a patient

7. Findings that shed new light on the possible pathogenesis of a disease or an adverse effect

All manuscripts submitted to *Journal of Medical Case Reports *will be subject to immediate screening by the in-house editorial team, and appropriate manuscripts will be sent for peer review. Accepted articles will be published online and indexed in PubMed immediately upon acceptance. For more information on the journal policies, please refer to the journal website [3].

We hope you will join us in this exciting, new venture, and submit your next case report to *Journal of Medical Case Reports*.

## Competing interests

MK is the Editor-in-Chief of *Journal of Medical Case Reports *and CH is an employee of BioMed Central, which publishes the journal.
